# Prevalence of canine heartworm (*Dirofilaria immitis*) disease in dogs of central Portugal

**DOI:** 10.1051/parasite/2014003

**Published:** 2014-02-19

**Authors:** Ana Luísa Vieira, Maria João Vieira, João Manuel Oliveira, Ana Rita Simões, Pablo Diez-Baños, Juan Gestal

**Affiliations:** 1 Catedrático Jefe del Servicio de Medicina Preventiva y Salud Pública del Hospital Clínico Universitario, Facultad de Medicina y Odontología, Universidade de Santiago Compostela España , Coordinador del Grupo de Santiago de Compostela del Centro de Investigación Biológica en Red de Epidemiología y Salud Pública (CIBERESP), y del grupo de Epidemiología del Instituto de Investigación Sanitaria (IDIS) de Santiago de Compostela 15782 Santiago Compostela Spain; 2 Catedrático de Universidad. Sanidad Animal. Grupo Investigación Sanidad Animal de Galicia (INVESAGA). Animal Pathology Department, Parasitology and Parasitic Disease. Facultad de Veterinaria de Lugo, Universidad de Santiago de Compostela 27002 Lugo España; 3 Clinicão – Hospital Veterinário 3080 Figueira da Foz Portugal; 4 EUVG – Escola Universitária Vasco da Gama 3000 Coimbra Portugal

**Keywords:** *Dirofilaria immitis*, Prevalence, Dog, Knott, ELISA, Portugal

## Abstract

The aim of the present study was to determine the prevalence and risk factors concerning *Dirofilaria immitis* infection in dogs from Figueira da Foz, located in the central region of Portugal. In the period between November 2009 and January 2011, 304 blood samples were obtained from dogs over 1 year of age, with no previous history of heartworm prevention or diagnosis. Every blood sample was analyzed using varied laboratory techniques (direct microscopic evaluation of a fresh blood sample, the modified Knott technique, and the ELISA antigen detection test – IDEXX Snapp®). In the samples in which microfilaremia was detected, a histochemical technique using acid phosphatase staining was applied to identify the species of microfilariae. A total prevalence of 27.3% (83 out of 304) was found. We also found that 73.5% of all positive cases (61 out of 83) were microfilaremic, and 26.5% were occult infections (22 out of 83). By means of a histochemical technique *Dirofilaria immitis* was identified in 96.7% of microfilaremic samples. A multivariate model allowed us to identify the following risk factors for the presence of heartworm disease: age between 4 and 9 years, dogs living in a rural environment, large breed dogs, and living outdoors. This study shows for the first time the high prevalence of heartworm disease in a central area of Portugal and emphasizes the importance of systematic screening for this disease, as well as the need to prevent it in dogs in this area.

## Abstract (fr)

Le but de cette étude était de déterminer la prévalence et les facteurs de risques de *Dirofilaria immitis* chez les chiens de Figueira da Foz, ville située dans la région centrale du Portugal. Entre Novembre 2009 et Janvier 2011, 304 échantillons de sang ont été prélevés chez les chiens de plus d’un an, sans antécédent de diagnostic ou prévention de *Dirofilaria immitis*. Chaque échantillon a été analysé en utilisant diverses techniques de laboratoire (évaluation directe au microscope d’un échantillon de sang frais, technique de Knott modifiée, test ELISA de détection des antigènes Idexx Snapp®). Pour les échantillons dans lesquels la présence de microfilaires a été détectée, une technique histochimique utilisant une coloration à la phosphatase acide a été utilisée pour identifier les espèces de microfilaires. Une prévalence totale de 27,3 % (83 sur 304) a été trouvée. Nous avons également constaté que 73,5 % de tous les cas positifs (61 sur 83) avaient des microfilaires, et que 26,5 % étaient des infections occultes (22 sur 83). Par une technique histochimique *Dirofilaria immitis* a été identifié dans 96,7 % des échantillons avec des microfilaires. Un modèle multivarié a identifié les risques suivants pour la présence de dirofilariose canine : âge entre 4 et 9 ans, vie en milieu rural, grande taille, vie à l’extérieur. Cette étude montre pour la première fois la forte prévalence de dirofilariose dans une zone centrale du Portugal et souligne l’importance du dépistage systématique de cette maladie ainsi que la nécessité de la prévenir chez les chiens de cette région.

## Introduction

*Dirofilaria immitis* (Leidy, 1856) [18] is a parasitic nematode infection responsible for canine and feline cardiopulmonary dirofilariasis. It is also the causal agent of human pulmonary dirofilariasis. It is a zoonotic parasitic disease mainly located in temperate, tropical, and subtropical areas of the world [27, 30]

Different species of culicid mosquitoes belonging to the genera *Culex, Aedes*, and *Anopheles*, among others, have been implicated in the transmission of this parasite, allowing for its intermediate stage to complete its life cycle [6, 30]. Weather is a critical factor because of climate requirements (high relative humidity, and higher than 15 °C average temperature) of the intermediate hosts [28].

Heartworm infection is a severe and life-threatening disease. The pathophysiological response to heartworm infection is mainly due to the presence of adult worms of *Dirofilaria immitis* in the pulmonary arteries and right ventricle of the heart [8, 17]. The worm numbers, host immune response, duration of infection, and host exercise levels determine the severity of the cardiopulmonary pathology [16, 28]. Furthermore, the symbiotic relationship with bacteria of the genus *Wolbachia* (Rickettsiaceae) stimulates an inflammatory response from the host’s immune system, amplifying disease severity [29].

Clinical manifestations include cough, dyspnea, weight loss, poor exercise tolerance, weakness, hemoptysis, cyanosis, and congestive heart failure [7, 17].

Canine heartworm infection is preventable and chemoprophylaxis is a priority in heartworm endemic areas. One of the goals of prevention is to reduce the reservoir population and decrease the prevalence of infection among unprotected dogs. The macrocyclic lactones (ivermectin, milbemycin oxime, moxidectin, and selamectin) are the most commonly used chemoprophylaxis agents: they are safe, effective and are administered either in oral, topical, or parenteral formulations at monthly or six-month intervals [19, 21, 22]. These drugs are effective against *Dirofilaria immitis* third-stage larvae (L3) and L4, which have developed within the previous 30 days, and thus prevent disease caused by adult worms [4, 13]. Prevention should start before the mosquito season in spring and should be continued until late fall [14]. Before starting a prophylactic regime, all mature dogs should perform a diagnostic test at least 6 months after the last administration of one of the macrolide compounds [14, 22].

The treatment of heartworm infection is neither simple nor safe in itself. The American Heartworm Society recommends the melarsomine dihydrochloride three-injection protocol (2.5 mg/kg followed 1 month later by two similar treatments 24 h apart) instead of the two-injection protocol of 2.5 mg/kg 24 h apart, because the former is safer in terms of thromboembolism and shock, and has a higher efficacy [23, 46, 47]. Recent studies suggest that the administration of both ivermectin and doxycycline for several months prior to melarsomine dihydrochloride or possibly even without melarsomine, will eliminate adult heartworm with lower risk of severe thromboembolism than melarsomine alone and will block transmission of the parasite [23, 24].

The presence of *Dirofilaria immitis* in dogs supposes a risk for the human population. Epidemiological, molecular, and immunological studies as well as clinical practice have discovered an increasing number of countries in which clinical cases of human dirofilariasis are reported, while seroepidemiological studies suggest that humans frequently become infected with *Dirofilaria spp.* at an early age. This information has changed the picture of human dirofilariasis from a sporadic to an emerging disease [20, 41]. In Portugal, a case of human ocular dirofilariasis was reported [15], and two cases of *D. immitis* infection in humans were reported in Portugal as a consequence of surgical removal and histological examination of larval lung nodules [1].

In the human host, *Dirofilaria immitis* is the causative agent of pulmonary dirofilariasis, and in many cases produces benign pulmonary nodules usually identified incidentally by chest radiography in asymptomatic patients, which can initially be misidentified as malignant tumors [12, 30–32, 44]. *Dirofilaria immitis* worms were also found in cranial, hepatic, intraocular and mesenteric adipose tissue, testicular arteries, and conjunctival regions [20, 41]. The clinical importance of human dirofilariasis was associated almost exclusively with iatrogenesis derived from surgical interventions to remove pulmonary nodules [20, 41].

The epidemiological status of dirofilariasis is currently undergoing rapid evolution. In spite of efforts made to prevent and control the infection in dogs, disease prevalence is rising in endemic areas as well as spreading into new areas reported as dirofilariasis-free until recently [40]. Factors such as global warming, changes in vectors’ seasonal population dynamics, animal circulation between countries, worm burden, and the age and immune response of the host, may play a role in the current geographical spread of the disease [10, 30]. In Europe, the highest prevalence has been reported in Mediterranean countries such as Italy, France, Greece, and Spain [30].

In the present study we will analyze the current prevalence and the seroprevalence of *Dirofilaria immitis* in dogs living in the central area of Portugal and explore some of the epidemiological conditions that may increase the infection.

## Materials and methods

### Study area, animals, and sample collection

The study was conducted in the county of Figueira da Foz, a central region of Portugal, in 18 contiguous parishes (379-km^2^ area) located near the Atlantic coast and crossed by the Mondego river. The climate is temperate. Winter temperatures range between 7 °C and 14 °C, rarely reaching below 0 °C, while in summer the temperatures range between 15 °C and 25 °C, sometimes exceeding 35 °C during heat waves. The average annual temperature is around 15 °C, while the mean annual precipitation is about 600 mm. The climate and socioeconomic life in the region with farming activity (rice culture) provide suitable conditions for development of culicid mosquitoes, vectors of Dirofilaria.

**Figure 1.**
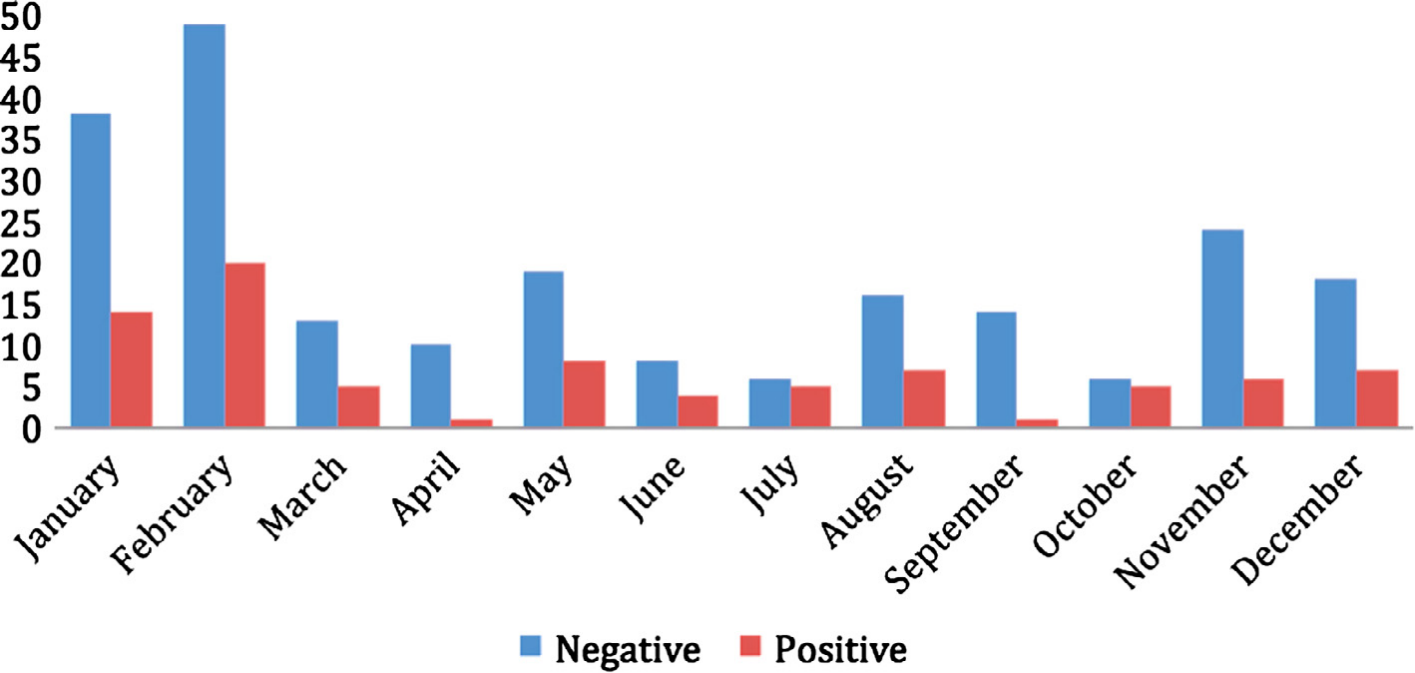
Distribution of heartworm results in investigated dogs according to months.

This study was carried out from November 2009 to January 2011, and 304 dogs (140 males and 164 females) were sampled: 247 animals were privately owned dogs which had been taken to the veterinary clinic for a routine health examination and 57 animals came from an animal rescue shelter (Animal Protection Association of Figueira da Foz), throughout the whole year (Figure 1).

Animals were selected to fulfill the following criteria: (1) native animals that had never been moved outside the study area according to the owners or the shelter’s representative, (2) animals more than 12 months old, (3) animals not submitted to chemoprophylaxis against heartworm, (4) animals with no past history or diagnosis of heartworm infection, and (5) owners who were willing to participate in this survey and authorized it.

The clinical signs and a detailed history (sex, age, weight, breed, hair length, utilization, indoor/outdoor status, and geographical origin) were obtained together with a complete physical examination looking for the presence of cardiopulmonary signs. This check-up included a complete cardiac and pulmonary auscultation, pulse evaluation, capillary refill time, jugular distension, and abdominal palpation.

A minimum of 5 mL of blood was collected from the jugular vein of all 304 dogs. Of this blood volume, at least 3 mL were collected in tubes with anticoagulant and at least 2 mL in tubes without anticoagulant and stored under refrigeration until analysis.

This study was approved by the Ethics Committee of the Clinicão – Veterinary Hospital and all of the dogs’ owners signed an informed consent document.

### Antigen detection

All samples of canine blood were tested for detection of *Dirofilaria immitis* circulating antigen using a canine antigen test kit (Canine Heartworm Antigen Test Kit, IDEXX Laboratories, Germany) with sensitivity of 84% (78–89), specificity of 97% (84–100), and accuracy of 86% (81–90) [2]. The procedure was carried out according to IDEXX’s instructions. A sample was considered positive if it developed a more intense color than the negative control. Each test was considered valid if the positive control developed a distinct blue color and the negative control was transparent or developed a very faint color.

### Microscopic examination

Blood samples were processed on the day of collection. First, fresh blood from each sample was examined under a microscope to determine the presence of microfilariae by the thick drop technique. A modified Knott test was also performed on all samples. Blood was hemolyzed with 9 mL of 2% formalin and centrifuged at 1500 rpm for 5 min. The supernatant was then decanted, and the sediment was stained with a drop of methylene blue 0.1% [9].

For species determination, microfilaremic-positive samples were analyzed using histochemical staining bases with different acid phosphatase activity using a commercial kit test (Leucognost SP^©^, Merck, Darmstadt, Germany) according to Peribánez et al. [36]. Microfilariae of *D. immitis* show two acid phosphatase activity spots localized around the anal and excretory pores, whereas *D. repens* shows only one acid phosphatase activity spot localized around the anal pore and microfilariae with enzyme activity throughout the body, shown to be *Dipetalonema reconditum* [36].

### Survey

From the 304 dogs used in the present study, 46.1% were males and 53.9% were females; 41.8% were pure breed and 58.2% were mixed breed; 37.8% were small breed, 39.8% were medium breed and 22.4% were large breed; 52% were short-haired, 28% were medium-haired and 20% were long-haired.

According to their habitats, 35.9% of the dogs were kept indoors and 64.1% remained outdoors; 56.9% lived in a rural environment and 43.1% lived in an urban environment, 80.9% had an owner and 19.1% came from an animal shelter.

Concerning their age, 26.3% of the dogs were between 1 and 3 years old, 42.7% were between 4 and 9 years old, and 31% were over 10 years old. As for their use, 69.3% were kept as pets, 10.1% were watchdogs, and 20.6% were hunting dogs.

### Statistical analysis

The data were analyzed using the SPSS^©^ Base 18.0 software for *Windows* ^©^ [42]. The chi-square test was performed to compare proportions when the variables were categorical.

A multivariate analysis allowed us to identify the risk factors for the presence of heartworm disease in this population. This multivariate analysis included those independent variables that presented a statistical significance of less than 0.2 in the bivariate analysis. The independent variables with the highest level of statistical significance were successively eliminated from the original model, provided that the coefficients of the principal variables of exposure changed by no more than 10%.

In all cases, the significance level was established at *p* < 0.05.

## Results and discussion

The information on the distribution and prevalence of *Dirofilaria immitis* in Portugal is incomplete. In the early 1990s, a study showed that canine heartworm infection was prevalent in several southern regions in Portugal, including Ribatejo (16.7%), Alentejo (16.5%), and Algarve (12%). The island of Madeira had the highest prevalence, with 30% of the dogs tested being positive for *Dirofilaria immitis* microfilaremia [1]. Prevalence may be underestimated because it was estimated solely as infection with heartworm microfilariae [7]. In another study from 2011, the overall canine heartworm prevalence in the north and central regions of Portugal was 2.1%, with the highest prevalence found in Aveiro (6.8%) and Coimbra (8.8%) [7]. In 2012, a study showed a prevalence of 3.6% in healthy Portuguese dogs and 8.9% in canine vector-borne disease (CBVD)-suspected Portuguese dogs. In this study the central region had a prevalence of 0.9% in healthy dogs and 7.4% in CBVD-suspected dogs. The region showing the highest prevalence was Madeira, with 40% in healthy dogs [7].

In the current study we demonstrate for the first time the existence of canine heartworm infection in Figueira da Foz (a central region of Portugal that belongs to Coimbra district) with 83 out of 304 dog samples analyzed testing positive in at least one diagnostic test, thus resulting in a prevalence of 27.3% (CI_95%_: 22.4%–32.7%). This value is higher than the previously estimated prevalence for Coimbra district and Portugal. Only the island of Madeira showed a similarly high prevalence.

The number of dogs that tested positive for *D. immitis* in one or more diagnostic tests was variable according to the techniques considered (Table 1). The prevalence values of *Dirofilaria immitis* were 19.4% (59/304) by the direct smear test, 20.1% by the modified Knott technique (61/304), and 25.7% (78/304) by the Snapp® IDEXX test. The resulting sensitivities were therefore 71.7% for the direct smear test, 73.5% for the modified Knott technique and 94% for the Snapp® IDEXX test. Specificity was 100% for the three techniques employed.

**Table 1.** Comparison of results of different diagnostic techniques for heartworm.

	Direct smear test	Modified Knott test	Snapp® IDEXX test	Total
Positive	59	61	78	83
Negative	245	243	226	221
Total	304	304	304	304
Prevalence (%)	19.4	20.1	25.7	27.3

The different sensitivities among the techniques could be explained by the fact that microfilariae-detecting techniques are not able to detect occult infections (amicrofilaremic infections). These infections could arise due to several causes: low parasite burdens, prepatent infection by young adults, infections by aging adult females with impaired fertility, infections with male-only parasites, or immune response from the host against microfilariae. The resulting percentage of occult infections was 26.5%. This high percentage of occult infections is not uncommon and was previously reported by several studies [23, 25, 39].

Five of the positive (6%) samples tested negative when using the Snapp^©^ IDEXX test but tested positive on the modified Knott test and were identified as belonging to *D. immitis* species by histochemical staining (acid phosphatase activity). Many studies have reported that commercial serological kits have low sensitivity when parasite burdens are low (one to five *Dirofilaria immitis* adult females), when the worms show low fertility, when the presence of microfilariae persists for 1 to 3 years after the death of adult females, prepatent infection or only male infection [23]. This could explain the result of five positive dogs (6%) that were microfilariae-positive and antigen-negative [35–37].

The samples of the 61 microfilaremic dogs underwent histochemical staining and 59 (96.7%) of them revealed acid phosphatase distribution matching with microfilariae of *D. immitis* (two distinct bright red bands of acid phosphatase activity on the excretory and anal pores specific to *D. immitis*). The acid phosphatase activity offers an easily observable and reliable method for differentiation of microfilariae [36, 49].

In regard to the diagnostic method, the McNemar statistical test allowed us to compare the different diagnostic techniques. The direct smear test and the modified Knott technique showed the same ability to perform the diagnosis, with no statistical differences (*p* = 1) and a high level of agreement with the dogs’ status of *Dirofilaria immitis* infection (Kappa = 0.781 and 0.801, respectively). The Snapp IDEXX® test demonstrated a much higher diagnostic capability, with a substantial level of agreement (Kappa = 0.958).

The following risk factors were therefore determined: dogs aged between 4 and 9 years old (37.8%), medium breed dogs (34.7%), dogs living in a rural environment (38%), dogs living outdoors (37.7%), watchdogs (52.2%), and hunting dogs (36.2%) (*p* < .05), whereas no significant differences were found related to ownership, pure versus mixed breed, sex or hair length.

A multivariate model identified the following risk factors for the presence of heartworm disease: age between 4 and 9 years (OR = 1.65; CI_95%_: 1.21–2.25; *p* < .01), dogs living in a rural environment (OR = 15.29, CI_95%_: 1.93–121.35; *p* < .05), large breed dogs (OR = 14.31; CI_95%_: 1.30–157.34; *p* < .05), and living outdoors (OR = 37.02; CI_95%_: 3.39–403.91; *p* < .001).

Estimation of canine heartworm prevalence by sex yielded inconsistent results. No significant differences by sex were reported in some studies [11, 43, 48]. On the other hand, some other authors [26, 28, 45, 49] have reported significantly higher prevalence in male dogs. In this study**,** there was no statistical difference between the sexes. For many authors, age is an important risk factor. The infection risk for dogs will probably remain stable throughout life and the probability of acquiring infection with *Dirofilaria immitis* is undoubtedly related to the increasing length of the period of exposure to the mosquitoes. Therefore, older dogs have higher prevalence of dirofilariasis than younger dogs [28, 49, 50]. We found the highest prevalence in animals between 4 and 9 years old (37.8%) and the lowest in animals under 3 years old (14.1%). In the present study, larger dogs showed higher prevalence than small dogs. In agreement with other studies, larger dogs seem to be more attractive for mosquitoes and spend more time outdoors [28, 48].

In Portugal there are 41 identified species of mosquitoes. The most abundant (92%) are *Anopheles atroparvus, Culex pipiens, Culex theileri*, and *Aedes (Ochlerotatus) caspius*, and they are broadly distributed among the 18 districts of Portugal [33]. Osório et al. demonstrated that the most common species of mosquitoes in the district of Coimbra were *Culex pipiens* (57%), *Culex theileri* (33.7%), and *Ochlerotatus caspius* (6.2%) [33]. In another study conducted in Portugal, Osório et al. found that both *Culex theileri and Ochlerotatus caspius species* fed on both hosts: *Canis familiaris* and *Homo sapiens* [34]. *Culex pipiens* is a natural and efficient vector of *Dirofilaria immitis* with a host-feeding pattern that includes humans, and is therefore of particular concern for animal and public health [3, 5, 34]. *Aedes caspius* was also described as a vector of *Dirofilaria immitis* [6]. In Portugal, the presence of larval forms of *Dirofilaria immitis* in mosquitoes of the species *Culex theileri* was found in Funchal [38] and in the area of Comporta, Alcácer do Sal [15, 37].

In this study, living in a rural environment and living outdoors are important risk factors and the activity of the dog is also a relevant health risk. Hunting dogs and watch dogs show higher prevalence. This influence is presumably due to vector exposure rates as they have a greater chance of being bitten by mosquitoes [28, 48, 49, 50]. No significant difference was found in the infection prevalence between owned or stray dogs. This is probably due to the lack of prophylaxis and public knowledge about the disease [49].

No significant difference was found in the results throughout the year. Although the period of transmission is restricted to a period of the year because of the relationship of the life of the vector with the weather, the long parasite development in dogs (7–9 months) and the fact that many of the infected dogs are asymptomatic for months or years makes it difficult to establish a curve during the year for prevalence [23] (Figure 1). We found that 64% of the *Dirofilaria immitis*-positive dogs were asymptomatic, with the risk of being silent carriers, which reinforces the importance of screening all dogs that live in Figueira da Foz that have not started any prevention strategy for *Dirofilaria immitis* yet.

The most common physical examination findings in this study were cough (38%), low body condition index (38.2%), increased capillary repletion time (66.7%), cardiac (48.8%), and pulmonary (66.6%) auscultation abnormalities, cardiac murmur (48.7%), and abdominal distention (48.1%). All these findings showed a significantly higher prevalence (*p* < 0.05). These physical examination findings are compatible with the pathophysiology of dirofilariasis disease [20, 39, 47].

The present study provides evidence that dogs in Figueira da Foz, Portugal, are at risk of becoming infected with *Dirofilaria immitis*. Given the impact on animal and human health, it is advisable that this infection should be included in the differential diagnosis of canine cardiopulmonary disease in the central area of Portugal. The epidemiological knowledge of human dirofilariasis in Portugal is still scarce. In Portugal, a case of ocular dirofilariasis in a human was reported [15], as well as two cases of *D. immitis infection* in humans as a consequence of surgical removal and histological examination of larval lung nodules [1]. Therefore, more studies are needed to understand the current situation better.

Continuing veterinary education and developing awareness will possibly avoid the spread of this parasitic disease throughout the country; for instance, by improving diagnostic and preventive measures against the vectors. This is particularly true given the possible lack of symptoms in microfilaremic animals and long incubation periods during which the animals are able to infect mosquitoes. The finding of positive microfilaremic but asymptomatic animals highlights the potential risk that exists. The presence of asymptomatic and microfilaremic animals in the present work demonstrates this potential risk.

Finally, this study is expected to give veterinary and public health authorities an increased awareness about the data concerning *Dirofilaria immitis* in the central area of Portugal, a situation that can be extended to other regions of the country where the information is also missing, thus contributing to the establishment of future control programs.

## Conflict of interest statement

We declare that we have no conflict of interest.
